# Creation of Stable Heterothallic Strains of Komagataella phaffii Enables Dissection of Mating Gene Regulation

**DOI:** 10.1128/MCB.00398-17

**Published:** 2017-12-29

**Authors:** Lina Heistinger, Brigitte Gasser, Diethard Mattanovich

**Affiliations:** a Christian Doppler Laboratory for Innovative Immunotherapeutics at Department of Biotechnology, BOKU-University of Natural Resources and Life Sciences Vienna, Vienna, Austria; b Department of Biotechnology, BOKU-University of Natural Resources and Life Sciences Vienna, Vienna, Austria

**Keywords:** Komagataella phaffii, *MAT* genes, *MAT* locus, Pichia pastoris, heterothallic strains, mating, mating type, pheromone receptor, sporulation, yeasts

## Abstract

The methylotrophic yeast Komagataella phaffii (Pichia pastoris) is homothallic and has been reported to switch mating type by an ancient inversion mechanism. Two mating-type (*MAT*) loci include homologs of the *MAT***a** and *MAT*α transcription factor genes, with the expression from one locus downregulated by telomere position effects. However, not much is known about mating gene regulation, since the mixture of mating types complicates detailed investigations. In this study, we developed K. phaffii strains with stable mating types by deletion of the inverted-repeat region required for mating-type switching. These heterothallic strains retain their ability to mate with cells of the opposite mating type and were used to further elucidate mating gene regulation. Functional analysis of *MAT* mutant strains revealed the essential role of *MAT***a***2* and *MAT*α*1* in diploid cell formation. Disruption of *MAT***a***1* or *MAT*α*2* did not affect mating; however, in diploid cells, both genes are required for sporulation and the repression of shmoo formation. The heterothallic strains generated in this study allowed the first detailed characterization of mating gene regulation in K. phaffii. They will be a valuable tool for further studies investigating cell-type-specific behavior and will enable in-depth genetic analyses and strain hybridization in this industrially relevant yeast species.

## INTRODUCTION

Mating, as the sexual reproduction of yeasts, is initiated by the mutual recognition of haploid cells of opposite mating types (**a** and α), which initiates a regulatory cascade leading to cell fusion and the formation of a diploid cell. Generally, the mating type of a cell is determined by the expression of the so-called *MAT* genes. Mating is best characterized in the budding yeast Saccharomyces cerevisiae, where one active *MAT* locus includes either the *MAT*α or the *MAT***a** genes. Silent copies of both *MAT* variants, named *HML*α and *HMR***a**, enable the cells to undergo mating-type switching. Exchange of the *MAT* cassette at the active locus is induced by HO endonuclease cleavage. The introduced double-strand break is subsequently repaired by synthesis-dependent strand annealing using the silent copy of the opposite mating type as the template (1–3). Due to this mechanism, wild-type S. cerevisiae strains are classified as secondary homothallic (self-fertile), while most laboratory strains carry mutations in the *HO* gene, rendering them heterothallic (4). By definition, heterothallic species have strains of different mating types, and only cells of opposite mating types can undergo mating. This is also true for secondary homothallic species; however, cells can switch their mating types, which allows mating between cells of the same strain. In primary homothallic species, cells usually express the *MAT* genes of both alleles, which allows mating of every cell with every other cell (5–7).

In S. cerevisiae, Matα1 is responsible for the activation of α-specific genes, whereas Matα2 is responsible for the repression of **a**-specific genes in a haploid α cell. Expression of the **a**-specific genes is constitutively activated in the absence of Matα2 and does not require the *MAT***a** gene products. Mat**a**1, together with Matα2, acts as a repressor of haploid-specific genes in diploid **a**/α cells. The *MAT***a** locus also includes a nonfunctional *MAT***a***2* gene. However, its sequence is identical to the 3′ end of the *MAT*α*2* gene and is not related to the *MAT***a***2* gene required for mating in most pre-whole-genome duplication yeasts (7–10). The regulatory network found in S. cerevisiae differs significantly from the one found in a broad range of yeast species, like Candida albicans, Kluyveromyces lactis, or Schizosaccharomyces pombe, where the expression of **a**-specific genes requires activation by Mat**a**2. As in S. cerevisiae, Matα1 is responsible for activation of α-specific genes. However, Matα2 does not act as a negative regulator of **a**-specific genes in these yeast species (7, 11–13).

Mating partner recognition is mediated by the small peptide pheromones **a**-factor and α-factor, respectively, which are secreted by haploid cells. They are recognized by pheromone receptors on the surfaces of cells of the opposite mating type (Ste2 on **a**-type cells and Ste3 on α-type cells), and pheromone binding leads to the activation of a common response pathway and initiation of the mating process. Deletion of the surface receptor genes has been shown to abolish the mating factor response in S. cerevisiae, K. lactis, and Ogataea polymorpha (14–18).

**FIG 1:**
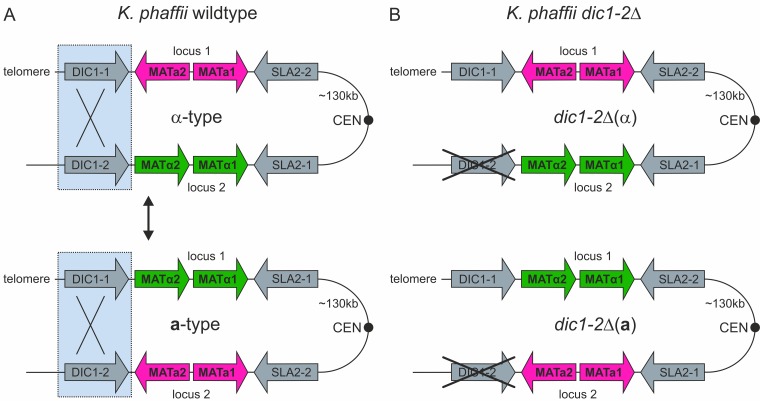
Mating-type loci in K. phaffii. (A) Mating-type loci in K. phaffii wild-type cells. The two *MAT* loci on chromosome 4 are flanked by inverted-repeat regions containing duplicated genes (*DIC1* and *SLA2*). Gene expression from locus 1 (silent locus) is downregulated by telomere position effects, while the genes in locus 2 (active locus) are expressed under mating conditions. The mating type of a cell is determined by the *MAT* allele in locus 2. Homologous recombination over the *DIC1*-containing “outer” repeat region leads to mating-type switching by inversion of the region between the two loci. (For further details see reference 22.) (B) Mating-type loci in heterothallic K. phaffii *dic1-2*Δ cells. Deletion of the *DIC1-2*-containing region flanking *MAT* locus 2 prevents mating-type switching.

The methylotrophic yeast Komagataella phaffii (often referred to by the old species name Pichia pastoris in the context of recombinant protein production) is a preferentially haploid yeast that is able to undergo mating and to form diploids under nitrogen limitation conditions. In contrast to S. cerevisiae, diploid cells are not stable and rapidly enter meiosis and sporulation (19). In K. phaffii, homologs of the *MAT* genes are in two different loci on chromosome 4, separated by approximately 135 kb of DNA sequence also containing the centromere. Both loci are flanked by inverted-repeat regions containing one (*DIC1*) and three (*SLA2*, *SUI1-1*, and *CWC25-1*) genes, respectively (20, 21). The organization of this region as it is found in the genome sequence of K. phaffii CBS2612 (unpublished data), as well as strain CBS7435, is shown in Fig. 1A. The mating type of haploid cells is determined by the positions of the *MAT***a** and *MAT*α genes in the genome. It has been suggested that the expression of the genes in locus 1 is downregulated by telomere position effects and that the *MAT* genes more actively transcribed from the second locus are responsible for the sexual identity of the cells (22, 23).

K. phaffii is a secondary homothallic species, and it has been proposed that mating-type switching takes place by chromosomal inversion of the whole region between the two *MAT* loci. This process likely involves homologous recombination of the 2.6-kb repeat region containing *DIC1* and leads to an exchange of the *MAT* genes in the two loci (22). However, the exact mechanism of mating-type switching is still unknown, as K. phaffii does not have an HO endonuclease homolog and no specific recombinases involved in switching have been identified so far. A similar inversion mechanism has also been described for O. polymorpha, where the mating type is determined by the repression of one of the *MAT* alleles by a centromere. More recently, Pachysolen tannophilus and Ascoidea rubescens have also been found to switch mating type by inversion of their *MAT* genes (17, 22, 24).

Due to the homothallic behavior of K. phaffii, cultures usually consist of an undetermined mixture of mating types, which complicates the detailed investigation of cell type regulation. The availability of heterothallic strains with a defined mating type would provide a valuable tool for the detailed characterization of mating-type-specific regulation and mating related processes in K. phaffii. Furthermore, such strains can facilitate the use of K. phaffii for classical genetic studies. As K. phaffii is a haploid pre-whole-genome duplication yeast, its genome includes paralogs of only a few genes, which simplifies genetic manipulation and studying the effects of mutations. Other, more specific applications are the breeding of strains to combine specific traits, as well as the analysis of industrially relevant phenotypic traits by quantitative trait locus (QTL) mapping, as applied to S. cerevisiae (25). Furthermore, K. phaffii is used as a host for the generation of libraries for optimization and selection of heteromultimeric proteins, like antibodies (26, 27). Compared to existing methods, the use of heterothallic strains for library mating should lead to an increase in the overall mating efficiency due to a loss of undesirable mating events between cells of the same strain.

In this study, the mating-type regulation of K. phaffii was investigated using a homothallic wild-type strain, as well as newly generated heterothallic strains. Phenotypic characterization of mating and transcript level analyses were used to elucidate the roles of the K. phaffii *MAT* genes in mating and sporulation.

## RESULTS

### *MAT* loci in K. phaffii strain CBS2612.

All the strains used in this study (Table 1) are based on the K. phaffii (P. pastoris) strain CBS2612. CBS2612 (NRRL Y-7556) is the type strain of the species K. phaffii (28). Sequence comparison between the strain CBS2612 and the strain CBS7435 used in previous studies showed 99% sequence identity (BLASTn) at both *MAT* loci (positions 1 to 8690 [locus 1] and 135093 to 143952 [locus 2] in CBS7435) (29, 30). Locus-specific PCR indicated a mixture of both mating types in the culture under all the cultivation conditions used.

**TABLE 1:** Strains used in the study

Strain	Genotype	Mating type	Resistance
CBS2612	Wild type	Mixed	
CBS2612 *mat***a***1*Δ	*mat***a***1*Δ::*loxP-ZeoR-loxP*	Mixed	Zeocin
CBS2612 *mat***a***2*Δ	*mat***a***2*Δ::*loxP-hphMX-loxP*	Mixed	Hygromycin B
CBS2612 *mat*α*1*Δ	*mat*α*1*Δ::*loxP-kanMX-loxP*	Mixed	Geneticin
CBS2612 *mat*α*2*Δ	*mat*α*2*Δ::*loxP-natMX-loxP*	Mixed	Nourseothricin
CBS2612 *mat***a***1* and -*2*Δ	*mat***a***1 mat***a***2*Δ::*loxP-ZeoR-loxP*	Mixed	Zeocin
CBS2612 *mat*α*1* and -*2*Δ	*mat*α*1 mat*α*2*Δ::*loxP-kanMX-loxP*	Mixed	Geneticin
CBS2612 *ste2*Δ	*ste2*Δ::*loxP-kanMX-loxP*	Mixed	Geneticin
CBS2612 *ste3*Δ	*ste3*Δ::*loxP-natMX-loxP*	Mixed	Nourseothricin
CBS2612 *dic1-2*Δ(α)	*dic1-2*Δ::*loxP-kanMX-loxP*	*MAT*α	Geneticin
CBS2612 *dic1-2*Δ(**a**)	*dic1-2*Δ::*loxP MAT*α-*loxP-natMX-loxP*^*a*^	*MAT***a**	Nourseothricin
CBS2612 *mat*a*1*Δ(α)	*dic1-2*Δ *mat***a***1*::*loxP-ZeoR-loxP*	*MAT*α	Zeocin
CBS2612 *mat*a*2*Δ(α)	*dic1-2*Δ *mat***a***2*::*loxP-hphMX-loxP*	*MAT*α	Hygromycin B
CBS2612 *mat*α*1*Δ(α)	*dic1-2*Δ *mat*α*1*::*loxP-kanMX-loxP*	*MAT*α	Geneticin
CBS2612 *mat*α*2*Δ(α)	*dic1-2*Δ,*mat*α*2*::*loxP-natMX-loxP*	*MAT*α	Nourseothricin
CBS2612 *mat***a***1*Δ(**a**)	*dic1-2*Δ *mat***a***1*::*loxP-ZeoR-loxP*	*MAT***a**	Zeocin
CBS2612 *mat***a***2*Δ(**a**)	*dic1-2*Δ *mat***a***2*::l*oxP-hphMX-loxP*	*MAT***a**	Hygromycin B
CBS2612 *mat*α*1*Δ(**a**)	*dic1-2*Δ *mat*α*1*::*loxP-kanMX-loxP*	*MAT***a**	Geneticin
CBS2612 *mat*α*2*Δ(**a**)	*dic1-2*Δ *mat*α*2*::*loxP-natMX-loxP*	*MAT***a**	Nourseothricin

a The *dic1-2*Δ(**a**) strain contains a *loxP-natMX-loxP* cassette located between *SLA2-2* and MATα*1* in *MAT* locus 1 and one additional *loxP* sequence in *MAT* locus 2 between and *SLA2-1* and *MAT***a***1*.

### Deletion of single *MAT* genes in the homothallic wild type.

As previously described, the mating type of K. phaffii is determined by the positions of the *MAT* genes in the genome, and telomere position effects might be responsible for downregulation of the genes present in the locus close to the telomere (22). This suggests that the presence of either *MAT***a***1* and -*2* or *MAT*α*1* and -*2* should be sufficient for mating-type-specific regulation in a haploid cell. To investigate the possibility of obtaining heterothallic strains by deletion of either the *MAT***a** or the *MAT*α genes, strains with single and double knockout of the individual *MAT* genes were generated. In all cases, the whole gene was replaced by an antibiotic selection marker, and the deletion was independent of the orientation of the *MAT* gene locus and thus of the mating type. This resulted in two possible combinations of strains in every mating and sporulation assay, which has to be considered for data interpretation. The ability to form diploids was evaluated by a standard mating procedure (19). Sporulation was induced by incubation on mating agar and analyzed by microscopy and diethyl ether extraction of the spores.

**FIG 2:**
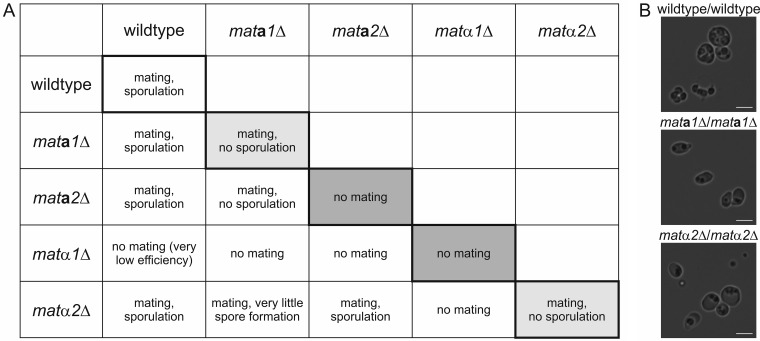
Effect of *MAT* gene deletions on mating and sporulation of homothallic K. phaffii cells. (A) Mating and sporulation phenotypes of *mat*Δ single mutants and the wild type. Crosses of genetically identical strains (shaded) indicated the essential roles of Mat**a**2 and Matα1 in mating, as well as the roles of Mat**a**1 and Matα2 in meiosis and sporulation. (B) Bright-field microscopy images of diploid wild-type/wild-type, *mat***a***1*Δ/*mat***a***1*Δ, and *mat*α*2*Δ/*mat*α*2*Δ cells under sporulation conditions. Bars, 5 μm.

The results of mating and sporulation experiments with all single-knockout strains and the wild type are shown in Fig. 2A. Representative mating plates for all combinations are shown in Fig. S1A in the supplemental material. As expected, K. phaffii wild-type cells were able to mate and sporulate under the conditions used. Also, all single *MAT* gene mutants except the *mat*α*1*Δ strain formed diploid cells and sporulated when mated with the wild type. Deletion of the *MAT*α*1* gene had a strong negative effect on the mating ability of the cells. No diploid cells could be obtained for any of the combinations with the other *mat*Δ strains, and hardly any colonies were obtained after mating with the wild type. Similarly, no diploid cell formation could be observed with *mat***a***2*Δ × *mat***a***2*Δ cells, although for this strain, mating was still possible with *mat***a***1*Δ and *mat*α*2*Δ cells, probably due to the presence of a higher number of *mat***a***2*Δ α-type cells carrying the deletion in *MAT* locus 1. This suggests that in K. phaffii *MAT*α*1*, as well as *MAT***a***2*, has a function in mating, although the effect of the *MAT*α*1* deletion was more pronounced, likely due to a higher proportion of α-type cells in the cultures. The mating behavior of *mat***a***1*Δ × *mat***a***1*Δ and *mat*α*2*Δ × *mat*α*2*Δ cells was comparable to that of the wild type. However, no viable spores could be isolated from the diploids obtained, indicating a role of *MAT***a**1 and *MAT*α2 in meiosis or sporulation. Microscopy of *mat***a***1*Δ/*mat***a***1*Δ and *mat*α*2*Δ/*mat*α*2*Δ cells after sporulation showed that the mutant cells were unable to form functional spores (Fig. 2B). Generally, many cells were observed to be deformed and contained varying numbers of granular structures, which might have resulted from failed spore formation. Of all the combinations of the single-deletion mutants, only diploid *mat*α*2*Δ/*mat***a***2*Δ cells yielded viable spores. Taking the previous results into account, these diploid cells could be formed only by *mat*α*2*Δ cells in **a**-type and *mat***a***2*Δ cells in α-type configurations, resulting in diploid cells expressing all 4 *MAT* genes from their two active *MAT* loci. As a result, all the diploid cells formed were able to sporulate normally. Additionally, *mat*α*1* and -*2*Δ and *mat***a***1 and -2*Δ double-deletion strains were tested for their mating abilities. As expected from the results of the single gene deletions, both double mutants were unable to mate and form diploids with each other. Generally, the data obtained are consistent under the assumption that a large majority of the cells in all the strains were α type. This strong imbalance toward the α mating type, and especially the more pronounced effect of the *MAT*α*1* mutation, suggests a low rate of mating-type switching during mating. Due to the undetermined mixture of mating types in the samples, no further information about *MAT* gene regulation could be obtained from these strains.

### Deletion of the pheromone surface receptor genes does not completely abolish self-mating.

The pheromone surface receptors Ste2 and Ste3 are required for mating partner recognition and activation of the signaling cascade leading to the induction of the mating process. In S. cerevisiae, their expression is mating type specific and regulated by the Mat transcription factors. In heterothallic yeasts, deletion of Ste2 or Ste3 completely prevents mating (16, 31, 32). In homothallic yeasts like K. phaffii, deletion of one possible receptor sequence should allow the cells to mate only as one possible mating type and result in a decreased overall mating efficiency.

**FIG 3:**
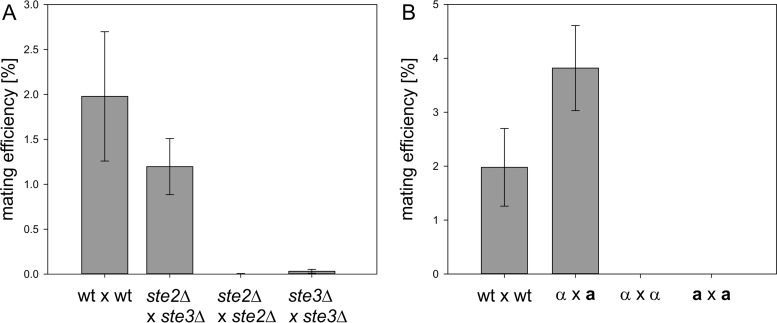
Mating efficiencies of pheromone receptor mutants and heterothallic *dic1-2*Δ strains. (A) Mating and self-mating efficiencies of *ste2*Δ and *ste3*Δ strains compared to the wild type (wt). Surface receptor mutants mated with reduced efficiency compared to the wild type. *STE2* and *STE3* deletion did not completely abolish self-mating. Averages of four independent mating experiments are shown. The error bars represent standard deviations. Student's *t* test showed significant differences (*P* < 0.05) between the mating efficiencies of the mutants and the wild type. (B) Mating and self-mating efficiencies of *dic1-2*Δ(**a**) and *dic1-2*Δ(α) strains compared to the homothallic wild type. No diploid cell formation between cells of the same mating type was observed with the heterothallic strains. Averages of 6 independent mating experiments are shown. The error bars represent standard deviations. Student's *t* test showed significant differences (*P* < 0.01) between the mating efficiencies of the mutants and the wild type.

As a possible strategy to obtain heterothallic strains and to investigate the effect of pheromone receptor deletions on the mating behavior of K. phaffii wild-type cells, *ste2*Δ (PP7435_Chr4-0893) and *ste3*Δ (PP7435_Chr3-1699) single mutants were generated. As for the *mat*Δ strains, the whole genes were replaced by antibiotic selection markers. The mating behavior of the strains was analyzed by the standard mating protocol. When *ste2*Δ cells were crossed with *ste3*Δ cells, the observed diploid cell formation was similar to that of the wild type. Interestingly, deletion of either Ste2 or Ste3 did not completely abolish mating of *ste2*Δ × *ste2*Δ and *ste3*Δ × *ste3*Δ cells. Images of representative mating plates are shown in Fig. S1B in the supplemental material. The results of the semiquantitative mating assays for quantification of the mating and self-mating efficiencies of the mutant strains are shown in Fig. 3A. The mating efficiency of the wild-type strain was found to be approximately 2%. Mating of *ste2*Δ cells with *ste3*Δ cells resulted in an efficiency of 1.2%. The self-mating efficiencies of *ste2*Δ × *ste2*Δ and *ste3*Δ × *ste3*Δ cells were 0.005% and 0.032%, respectively. Once formed, diploid *ste*Δ cells sporulated with an efficiency similar to that of the wild type.

### Deletion of the outer repeat region prevents mating-type switching.

Another possibility to obtain heterothallic K. phaffii strains is to prevent mating-type switching. It has been suggested previously that mating-type switching in K. phaffii takes place by homologous recombination of the outer inverted-repeat regions flanking both *MAT* loci, followed by an inversion of the whole region of chromosome 4 (22). So far, no specific recombinases or other factors required for this recombination have been identified. To prevent mating-type switching, strains with a 2,600-bp deletion of the entire outer repeat region of *MAT* locus 2, containing the second copy of the *DIC1* gene (*DIC1-2*; whole region, positions 140698 to 143262 in CBS7435 [29, 30]), were generated (Fig. 1B). In these mutants, only one *MAT* configuration could be detected by PCR, indicating that mating-type switching was no longer possible. However, all the clones obtained were *MAT*α [referred to here as *dic1-2*Δ(α)] [throughout this work, the mating type of strains is denoted by (**a**) or (α) and always refers to the *dic1-2*Δ genotype]. Therefore, **a**-type mating partners were generated by exchanging the *MAT*α*1* and *-2* genes in *MAT* locus 2 for *MAT***a***1* and *-2* by integration of the PCR-amplified sequences flanked by homologous regions for targeting. In the next step, *MAT*α*1* and *-2* were reintegrated at *MAT* locus 1 [named *dic1-2*Δ(**a**)] using the same approach. The absence of unwanted recombination events after the two integration and marker recycling steps was confirmed by PCR and sequencing. The mating and self-mating abilities of the *dic1-2*Δ mutants were investigated by qualitative and semiquantitative mating assays (Fig. 3B). Generally, the mating behavior of the mutants was similar to that of the wild-type and diploid cells, and viable spores could be obtained when *dic1-2*Δ(α) cells were crossed with *dic1-2*Δ(**a**) cells. No mating was observed between *dic1-2*Δ(α) and *dic1-2*Δ(α) or *dic1-2*Δ(**a**) and *dic1-2*Δ(**a**) cells, showing that deletion of the outer repeat region is sufficient to prevent mating-type switching. With an average mating efficiency of 3.8%, the mating efficiency of the mutants was about 2-fold higher than that of the wild type. In the heterothallic strains, mating is possible only between cells carrying different selective markers, avoiding the formation of diploids not able to grow on the selection plates used in the assay. Furthermore, a more balanced ratio between the mating types in the culture might be an additional cause of the increased mating efficiency.

### In heterothallic strains, mating genes are expressed in a mating-type-specific manner under mating conditions.

**FIG 4:**
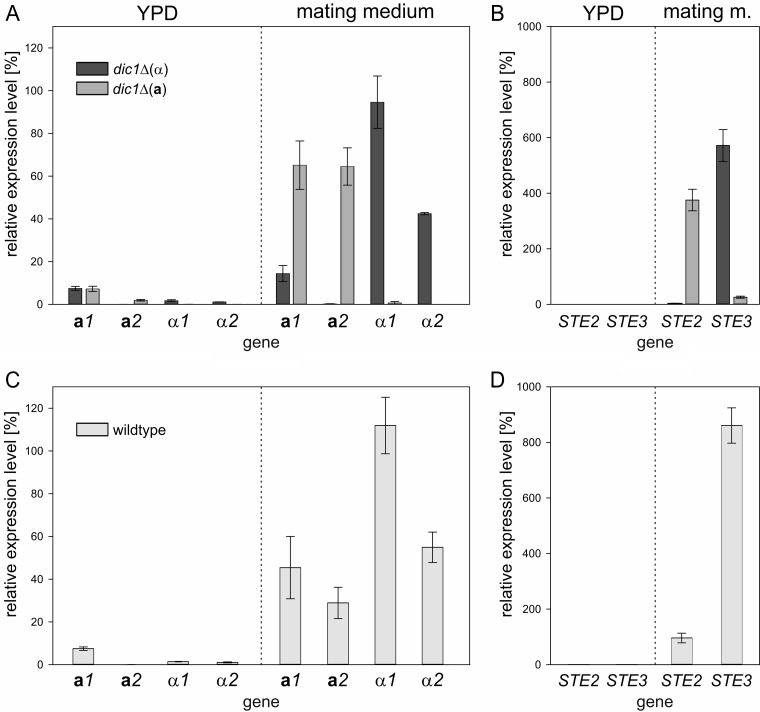
Transcript levels of mating genes under mating and exponential growth conditions. For transcript level analysis, cells were first grown in rich (YPD) medium to analyze gene expression during exponential growth and then shifted to nitrogen-free mating medium to induce mating gene expression. Transcript levels were normalized to *ACT1* expression. The error bars represent the standard deviations of three biological replicates. Student's *t* test showed significant differences in gene expression between the mating types and culture conditions (*P* < 0.05) (for details, see Tables S1 and S2 in the supplemental material). (A) *MAT* gene expression in *dic1-2*Δ(α) and *dic1-2*Δ(**a**) cells is induced under mating conditions in a mating-type-specific manner. (B) Mating-type-specific pheromone surface receptor gene expression in *dic1-2*Δ(α) and *dic1-2*Δ(**a**) cells. (C) Increased levels of all 4 *MAT* transcripts were detected in cultures of homothallic K. phaffii wild-type cells under mating conditions. (D) Induction of pheromone surface receptor gene expression in homothallic K. phaffii wild-type cells under mating conditions.

To learn more about the regulation of mating-relevant genes in **a**- and α-type strains of K. phaffii, the transcript levels of the *MAT* and pheromone receptor genes were determined by quantitative PCR (Fig. 4). Cells were grown in rich medium, followed by incubation in mating medium for 24 h to induce the expression of the *MAT* genes.

As expected, *MAT* gene expression was low under standard growth conditions, with *MAT***a***1* showing the highest expression at approximately 7.5% of that of the reference *ACT1*. This background expression was the same in both mating types, as well as the homothallic wild-type strain (Fig. 4A and C). After 24 h in mating medium, strong and mating-type-specific upregulation of gene expression could be observed. In **a**-type cells, *MAT***a***1* and *MAT***a***2* transcript levels were upregulated to similar levels, while the levels of the *MAT*α transcripts remained low. A mating-type-specific effect was also seen in α-type cells; however, the level of *MAT*α*1* expression was approximately 2-fold higher than that of *MAT*α*2*. Additionally, some increase of *MAT***a***1* transcript levels was also observed in α-type strains under mating conditions. The expression of the pheromone surface receptor genes (Fig. 4B) was also found to follow the same pattern of mating-type-specific upregulation of expression under mating conditions, with *STE2* expressed only in **a**-type cells and *STE3* upregulated to very high levels only in α-type cells.

Analogous to the cultures of the stable mating types, cultivation in mating medium also led to strong upregulation of *MAT* gene expression in cultures of the homothallic wild-type strain (Fig. 4C). As expected, the expression levels of *MAT***a***1* and *MAT***a***2* in samples from the mixed cultures were lower than in the pure **a**-type cultures. Interestingly, the *MAT*α transcript levels detected for the wild type were even slightly higher than in the α-type samples. For *STE*2 and *STE*3 (Fig. 4D), this effect was even more pronounced. Only weak induction of *STE2* expression and very strong induction of *STE3* expression to a level higher than that observed in pure α-type cultures were observed. These strong differences in transcript levels again indicated an unbalanced ratio of **a**- and α-type cells, with a strong bias toward the α mating type, in the cultures of the K. phaffii wild-type strain used.

### Single *MAT* deletions in stable mating types reveal *MAT* gene functions.

To further elucidate the roles of the single *MAT* genes in mating regulation, heterothallic strains with a disruption in one of the *MAT* genes were generated by clustered regularly interspaced short palindromic repeat (CRISPR)/Cas9-mediated integration. These strains were analyzed for their mating and sporulation phenotypes as described above.

**FIG 5:**
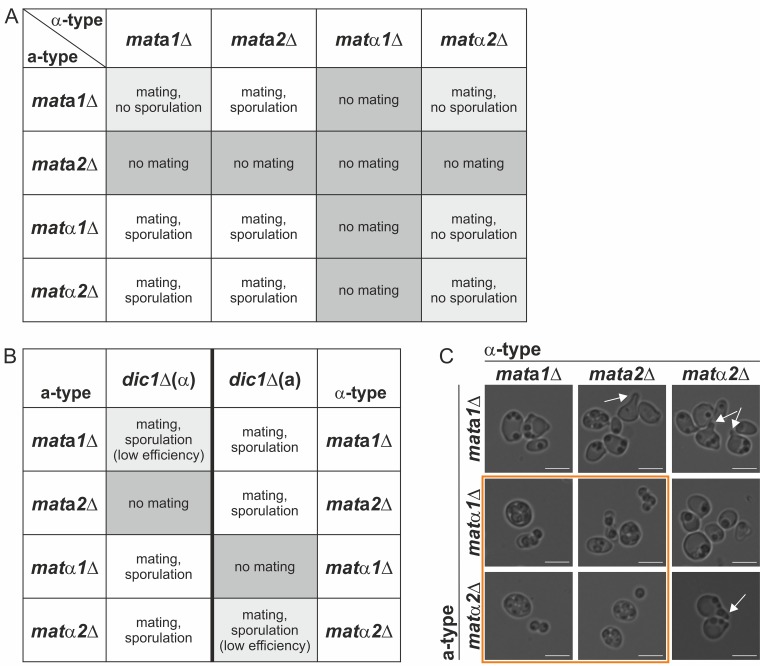
Disruption of single *MAT* genes in heterothallic strains revealed their roles in mating and sporulation. (A) Mating and sporulation phenotypes of combinations of all *dic1-2*Δ single *MAT* gene disruption strains. Mating was not possible in any of the crosses with *mat***a***2*Δ(**a**) and *mat*α*1*Δ(α) cells, confirming the essential roles of Mat**a**2 and Matα1 in diploid cell formation. Disruption of *MAT***a***1* in **a**-type cells or *MAT*α*2* in α-type cells resulted in a sporulation defect in diploid cells [reduced mating efficiency in *mat***a***1*Δ(**a**)/*mat***a***2*Δ(α) cells]. (B) Mating and sporulation phenotypes of *dic1-2*Δ single *MAT* mutants crossed with the unmodified *dic1-2*Δ strain of opposite mating type. (C) Bright-field microscopy images of diploid *MAT* mutant cells with stable mating types under sporulation conditions. Wild-type-like tetrads were formed by cells carrying the mutated genes in the silent *MAT* locus (inside the orange box). No mature spores could be observed in diploid *mat***a***1*Δ(**a**) and *mat*α*2*Δ(α) cells [only little spore formation in *mat***a***1*Δ(**a**)/*mat***a***2*Δ(α) cells]. The arrows indicate shmoo formation. Bars, 5 μm.

When two *dic1-2*Δ *mat*Δ strains were crossed, a phenotype could be observed only if at least one of the mutated *MAT* genes was located in the active *MAT* locus (Fig. 5A). Disruption of either *MAT***a***2* in *dic1-2*Δ(**a**) or *MAT*α*1* in *dic1-2*Δ(α) led to a complete loss of mating competence in combination with other *mat*Δ strains, as well as the respective wild-type strain of opposite mating type, which confirmed the essential roles of *MAT***a***2* and *MAT*α*1* in diploid cell formation. Surprisingly, a low number of diploid cells were obtained when *mat***a***2*Δ(**a**) cells were crossed with different *dic1-2*Δ(**a**) strains (see mating plates in Fig. S1C and D in the supplemental material), indicating a role of *MAT***a***2* in the repression of the α-type genes. However, no elevated levels of the *MAT*α*1* or *MAT*α*2* transcripts could be detected in the *mat***a***2*Δ(**a**) strain under mating conditions (see Table S3 in the supplemental material). A mutation in the active *MAT***a***1* or *MAT*α*2* gene did not have an effect on mating. However, the diploid cells obtained were unable to form viable spores, indicating a role of *MAT***a***1* and *MAT*α*2* in meiosis or sporulation. Interestingly, a small number of viable spores were formed when these *MAT***a***1* or *MAT*α*2* mutants were mated with the respective *dic1-2*Δ wild-type strain (Fig. 5B). Viable spores were also formed by *mat***a***1*Δ(**a**)/*mat***a***2*Δ(α) cells, indicating that the *MAT***a***1* background expression by the functional gene in locus 1 provided by the mating partner might be sufficient for sporulation in some of the cells. To a lesser extent, the same low expression level could cause sporulation in some diploids formed by *mat***a***2*Δ(**a**) cells and the unmodified *dic1-2*Δ(α) strain. Microscopy of diploid *mat***a***1*Δ(**a**) and *mat*α*2*Δ(α) mutants under sporulation conditions (Fig. 5C) showed irregularly shaped cells containing varying numbers of small granular structures. Some of the cells also formed shmoo-like extensions, indicating that both Mat**a**1 and Matα2 are required for repression of mating in diploid K. phaffii cells. With the exception of *mat***a***1*Δ(**a**)/*mat***a***2*Δ(α) cultures, where a small number of cells were found to form wild-type-like tetrads, no regularly sporulating cells could be observed in the combinations with *mat***a***1*Δ(**a**) or *mat*α*2*Δ(α) mutants. In the diploid cells combining two mutations in the silent *MAT* locus, most cells contained four large spores, and a large number of free spores were visible after cultivation on nitrogen-free plates for 3 days.

**FIG 6:**
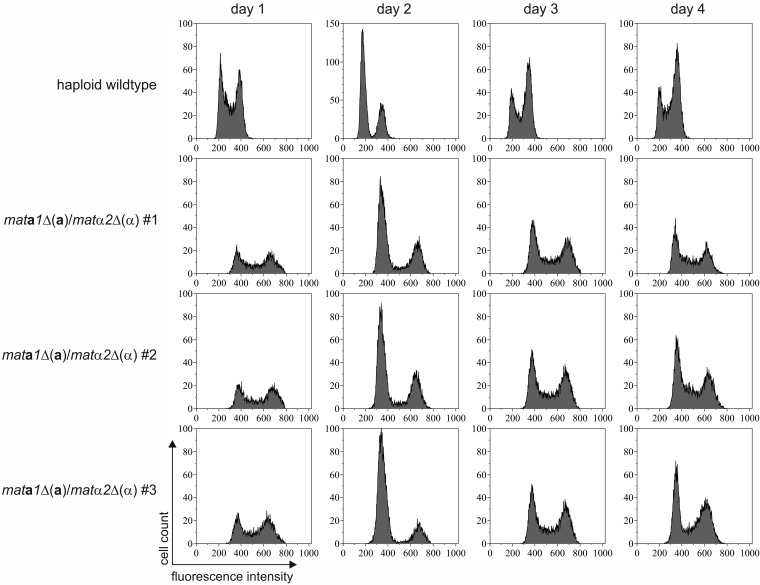
Stability of diploid *mat***a***1*Δ(**a**)/*mat*α*2*Δ(α) cells. Diploid cells and the unmated control were cultivated in YPD medium without selective pressure. The DNA content of the cells was analyzed by propidium iodide staining, followed by flow cytometry. The measured fluorescence intensity directly correlated with DNA content. The two peaks in the unmated wild-type sample represent G_0_/G_1_ (1N) and G_2_ (2N) phase cells. The peaks were shifted to higher fluorescence in diploid cells (2N and 4N). The DNA content of the diploid *mat***a***1*Δ(**a**)/*mat*α*2*Δ(α) cells remained constant for 4 days in culture. The results for three independent diploid clones and an unmated wild-type culture are shown.

Generally, diploid cells were able to divide and grow on rich medium. However, they remained diploid only as long as selective pressure was maintained. For the *mat***a***1*Δ(**a**)/*mat*α*2*Δ(α) cells, additional growth experiments showed that the mutants were able to grow on rich medium without antibiotics for selection for several days while maintaining a diploid state (Fig. 6).

The *MAT* gene transcript levels under mating conditions were analyzed by quantitative PCR (see Table S3 in the supplemental material). No large changes that could indicate direct activation or repression of *MAT* gene transcription by one of the other *MAT* gene products were observed. Some of the smaller differences in expression were found to be statistically significant; however, the biological relevance of such small changes in transcript level is hard to evaluate. Overall, the data suggest that there is no additional repression of the downregulated genes in locus 1 by the Mat proteins encoded in the active *MAT* locus and that mating-type regulation happens solely by position effects.

### Mata 2 and Matα1 are required for activation of mating-type-specific gene expression.

**FIG 7:**
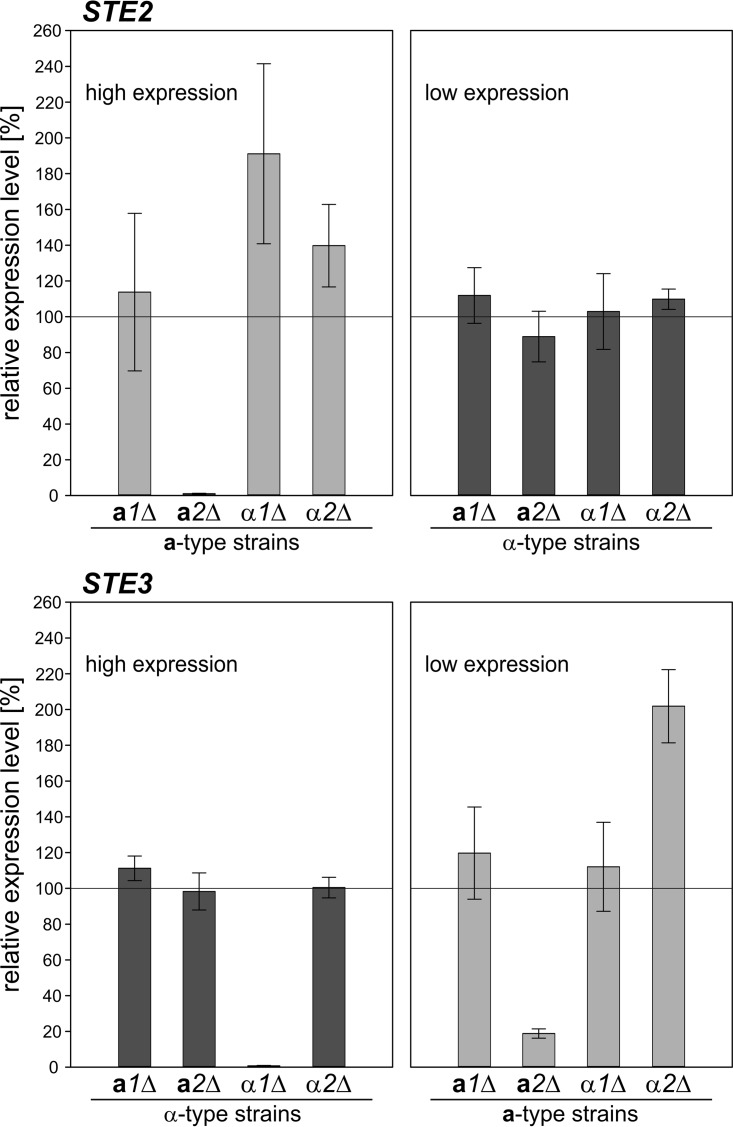
Relative transcript levels of *STE2* and *STE3* in *dic1-2*Δ *mat*Δ strains under mating conditions. The expression of *STE2* and *STE3* in the *dic1-2*Δ *mat*Δ strains was analyzed in nitrogen-free medium to induce mating gene expression. Gene expression was normalized to *ACT1* and is given relative to the expression in an unmodified *dic1-2*Δ strain of the same mating type. Note that absolute *STE2* levels were high in all **a**-type strains, whereas *STE3* was highly expressed in all α-type strains. The error bars represent the standard deviations of two biological replicates measured in triplicate. The differences from the respective *dic1-2*Δ strains were assessed using Student's *t* test. The *P* values are listed in Table S3 in the supplemental material.

Levels of *STE2* and *STE3* mRNAs were found to be reduced to noninduced levels in the absence of functional Mat**a**2 in **a**-type and Matα1 in α-type cells (Fig. 7). This indicated direct activation of pheromone receptor gene expression by Mat**a**2 and Matα1, depending on the mating type. An additional effect on low-level *STE3* transcription was observed in *mat***a***2*Δ(**a**) cells. The missing activation of mating-type-specific genes, such as *STE2* and *STE3*, may subsequently lead to the nonmating phenotype of *mat***a***2*Δ and *mat*α*1*Δ cells. Furthermore, no large changes in the background transcript levels of *STE2* and *STE3* were detected in the different mutant strains, indicating that the *MAT* gene transcripts are not directly involved in the repression of mating-type-specific genes of the opposite mating type.

## DISCUSSION

Wild-type K. phaffii strains are homothallic and undergo mating-type switching by homologous recombination and inversion of the chromosomal region between the two *MAT* loci (22). In contrast to S. cerevisiae, the K. phaffii genome does not encode an HO endonuclease homolog, and so far, no other specific endonucleases for the induction of mating-type switching that could be targeted for the generation of heterothallic strains have been identified. The same is true for O. polymorpha, which is also able to switch mating type by an inversion mechanism (17, 22). Generally, yeast mating requires the expression of mating-type-specific pheromones and corresponding surface receptors for mating partner recognition and activation of mating signaling (33). Deletion of the genes encoding the surface receptors on **a**- and α-type cells has been shown to prevent mating within a homothallic population of the methylotrophic yeast O. polymorpha, resulting in strains that behave like heterothallic strains (17). In our experiments, the deletion of the receptor homologs in K. phaffii could not completely prevent self-mating within a population of *ste2*Δ or *ste3*Δ cells. However, the observed mating efficiencies were approximately 100 times lower than the mating efficiency of *ste2*Δ × *ste3*Δ cells. Although the recognition of mating pheromones is crucial for efficient mating, it seems that in K. phaffii little diploid cell formation is possible even if one of the mating partners is missing the pheromone receptor of its respective mating type and should therefore be unable to sense the opposite mating pheromone. It is possible that the background expression of the remaining surface receptor gene is sufficient to allow some mating under favorable conditions, although the receptor does not correspond to the mating type of the cell. However, this would also allow autoactivation of the pheromone response pathway, because cells secrete mating factor that can subsequently be recognized by their own receptor. Such self-mating has not been observed in any of the other strains tested in this study.

The deletion of the whole homologous region flanking the active *MAT* locus required for mating-type switching allowed the generation of heterothallic K. phaffii strains. The strains obtained were able to mate with the wild type and cells of opposite mating type with good efficiency. Furthermore, no self-mating could be observed within the stable strains, showing that mating-type switching does not occur in these strains. Transcript level analysis showed that *MAT* gene expression was reduced to background level during exponential growth in rich medium. In a previous study, *MAT* gene expression was reported to be slightly higher under exponential growth conditions (22). *MAT***a***1*, especially, was found to be expressed at levels almost as high as the control *ACT1*, whereas in this study, *MAT***a***1* levels were below 10% of *ACT1* expression in all strains cultivated in rich medium. Furthermore, this background expression was reported to be dependent on *MAT* orientation for *MAT***a***2* and *MAT*α*2*, which could be confirmed, even though the transcript levels were extremely low. Upon cultivation on nitrogen-free medium, *MAT* gene expression was induced in a mating-type-specific manner. The same was true for the expression of the *STE2* and *STE3* genes encoding the pheromone receptors. In cultures of the homothallic wild-type strain, the *MAT*α*1*, *MAT*α*2*, and *STE3* transcript levels detected were found to be higher than in pure α-type cultures. This points to an additional activation of *MAT* gene expression in the presence of mating pheromone secreted by cells of opposite mating type. A similar pattern of gene regulation has been described for Candida lusitaniae and S. pombe, where mating-type-specific genes are expressed only under mating conditions and activation of the pheromone response pathway creates a positive-feedback loop for the activation of genes required for mating (34–37). Interestingly, regulation seems to be different in the more closely related methylotrophic yeast O. polymorpha, where it has been shown that *STE2* and *STE3* are constitutively expressed in mitotically growing cells (17).

The analysis of *MAT* gene mutants of the homothallic wild-type strain had already indicated that in K. phaffii the *MAT***a***2* and *MAT*α*1* gene products are essential for diploid cell formation, whereas functional *MAT***a***1* and *MAT*α*2* genes are required for the formation of viable spores. However, the mixture of mating types complicated further data interpretation, and the roles of the K. phaffii *MAT* genes were further analyzed using the newly generated heterothallic *MAT* mutants with defined mating types. Generally, there was no effect on the observed mating phenotype if the mutated gene was located in *MAT* locus 1. No diploid cells could be formed by **a**-type cells missing a functional *MAT***a***2* or α-type cells with a deletion of *MAT*α*1* in the active *MAT* locus. This effect was independent of the mating partner. Transcript level analysis showed a reduction of *STE2* and *STE3* transcripts to noninduced levels in the *mat***a***2*Δ(**a**) and *mat*α*1*Δ(α) strains, respectively, suggesting that in K. phaffii *MAT***a***2* and *MAT*α*1* are responsible for the activation of mating-type-specific gene expression. This mechanism of mating-type regulation differs from the one described for S. cerevisiae, where the expression of **a**-specific genes is constitutive in the absence of the Matα2 repressor, and corresponds to the transcriptional regulation described for other yeast species, like C. albicans, C. lusitaniae, S. pombe, and O. polymorpha (11, 12, 17, 34).

Although Mat**a**2 and Matα1 seem to have the same function in **a**- and α-type cells, respectively, deletion of one of the genes resulted in different phenotypes in the mating experiments performed with the homothallic *MAT* mutants. None of the combinations with the *mat*α*1*Δ strain resulted in diploid cell formation, whereas mating of the *mat***a***2*Δ strain with the *mat***a***1*Δ and the *mat*α*2*Δ strains was still possible. This difference in the mating phenotype could be explained by a higher proportion of α-type cells in the wild-type strain, which would place the *MAT***a***2* mutation in the silent *MAT* locus in the majority of cells. In that case, mating of a *mat***a***2*Δ strain would always be possible to some extent, as long as the mating partner had a functional *MAT***a***2* or *MAT*α*1* gene. Another indication that the wild-type strain was mainly *MAT*α came from transcript level analysis. In cultures of K. phaffii wild-type cells under mating conditions, *MAT***a** gene expression levels were lower than in stable **a**-type cells. At the same time, *MAT*α transcript levels were higher than in stable α-type cells. Additionally, very strong induction of *STE3* and only weak induction of *STE2* expression was observed in the wild type, pointing to a higher proportion of α-type cells in all of the cultures analyzed. The strong imbalance of mating types under mating conditions suggests a low rate of mating-type switching even under nitrogen starvation conditions. Theoretically, a high switching rate in the homothallic wild type should result in mating efficiencies similar to those obtained for the heterothallic strains, even if the starting culture consists mostly of one mating type. In O. polymorpha, cultivation in nitrogen-free medium was shown to induce inversion of the *MAT* locus, and mating efficiencies in cultures with only one mating type were found to be similar to efficiencies in mixed cultures (17, 22). Further experiments would be necessary to determine the rate of mating-type switching in K. phaffii and to analyze whether the large proportion of α-type cells in all the strains is a clonal effect, as all the strains were derived from the same wild-type strain, or whether there is a general preference for one mating type in mitotically growing cultures.

The disruption of *MAT***a***1* or *MAT*α*2* in the active *MAT* locus did not affect mating. Additionally, no strong changes in the *MAT*, *STE2*, and *STE3* gene expression levels could be observed in these mutant strains. Taken together, this suggests that in K. phaffii *MAT***a***1* and *MAT*α*2* are not required for the repression of the opposite mating type by either negatively regulating expression of the *MAT* genes in locus 1 or directly repressing mating-type-specific gene expression. However, diploid cells with a disrupted *MAT***a***1* or *MAT*α*2* in the active locus were unable to form viable spores, showing that the cells need the activities of both of the proteins to successfully undergo meiosis and sporulation. The same sporulation phenotype has been described for O. polymorpha (17) and in more detail for S. cerevisiae, where the Mat**a**1-Matα2 heterodimer is required for the repression of haploid-specific genes in diploid cells, which blocks mating and allows entry into meiosis (38). The observed shmoo formation of diploid cells of *MAT***a***1* and *MAT*α*2* mutants under sporulation conditions further supported the role of a Mat**a**1 and Matα2 complex acting together to repress haploid-specific genes in K. phaffii.

One interesting exception to the observed sporulation defect of the *MAT***a***1* and *MAT*α*2* mutants was viable spores formed with low efficiency by *mat***a***1*Δ(**a**)/*mat***a***2*Δ(α) and *mat***a***1*Δ(**a**) diploid cells in combination with the unmodified *dic1-2*Δ(α) strain. It is possible that although the active *MAT***a***1* gene was mutated, the relatively high background expression of *MAT***a***1* in the α-type configuration was sufficient for successful spore formation in some of the diploid cells. A possible role of Mat**a**1 under nonmating conditions and the transcriptional regulation leading to higher *MAT***a***1* expression from the silent *MAT* locus than for the other *MAT* genes still need to be investigated.

Overall, mating-type regulation in the methylotrophic yeast K. phaffii was found to be similar to the regulatory circuit described for other pre-whole-genome duplication yeasts. The functional characterization of *MAT* mutants showed that Mat**a**2 and Matα1 are essential for the activation of mating-type-specific gene expression in haploid cells, while in diploid cells, both Mat**a**1 and Matα2 are required for meiosis and sporulation. The heterothallic K. phaffii strains generated in this study allowed a first analysis of mating-type-specific gene expression under different conditions and will prove invaluable for further studies on mating regulation. Furthermore, they will be a useful tool, opening the way for detailed genetic studies of K. phaffii and enabling a variety of industrially relevant applications, such as the selection of desired traits by crossing of strains and the efficient generation of combinatorial libraries for the screening of complex multimeric proteins.

## MATERIALS AND METHODS

### Yeast strains and vectors.

All the K. phaffii strains used in this study (Table 1) were derived from the wild-type strain CBS2612 (28). For gene deletions, the target genes were replaced by an expression cassette encoding an antibiotic selection marker mediated by approximately 1,000 bp of homologous sequence for integration using the split-marker approach (39, 40). The knockout cassettes were generated using three different methods. A schematic of the strategies is shown in Fig. S2 in the supplemental material. Generally, they consisted of a homologous region A; two marker fragments, B and C, overlapping for approximately 440 bp; and a homologous region D. Fragments A and D were amplified from genomic DNA of K. phaffii CBS2612, while the marker fragments were amplified from plasmid vectors carrying the desired antibiotic marker gene. The zeocin resistance cassette (*ZeoR*) consisted of the Streptoalloteichus hindustanus *ble* gene (bleomycin resistance gene) under the control of the S. cerevisiae *TEF1* promoter and the Ashbya gossypii *TEF1* terminator. For the *mat***a***1*Δ and the *mat*α*1* and -*2*Δ strains, fragments A-B and C-D were joined in a second fusion PCR step. For the *mat***a***2*Δ, *mat*α*1*Δ, and *mat*α*2*Δ strains, fragments were amplified with primers carrying overhangs with fusion sites for Golden Gate cloning (41, 42, 47), and fragments A-B and C-D were assembled into two separate backbone 3 (BB3) vectors containing the required fusion sites. Before transformation, the split-marker-type knockout cassettes were PCR amplified from these vectors. For the *MAT***a***1* and -*2*, *STE2*, *STE3*, and *DIC1-2* deletion strains, as well as the *MAT***a***1* and -*2* and *MAT*α*1* and *-2* integrations, the required fragments were again amplified with primers containing overhangs for Golden Gate cloning and assembled into one backbone vector (BB3). In this case, the complete antibiotic marker gene was cloned as one sequence (fragment B-C), and the separate knockout cassettes with overlapping ends were subsequently amplified from the assembled vectors by PCR. All the primers used in this study are listed in Table S4 in the supplemental material. To mutate the single *MAT* genes in the *dic1-2*Δ strains, an integration cassette consisting of an antibiotic marker gene and a stretch of stop codons was integrated into the 5′ end of the coding sequence by using CRISPR/Cas9-mediated homology-directed repair. Homology templates were assembled into a BB3 Golden Gate vector and cut out by BpiI digestion before transformation. Human codon-optimized Cas9 (43) under the control of the constitutive K. phaffii *PFK300* promoter and a guide RNA targeting the integration site under the control of the *GAP* promoter and flanked by self-splicing ribozyme sequences (44) were expressed from an episomal plasmid vector. Correct gene replacement was verified by PCR with primers binding outside the homologous regions used for integration. All the marker cassettes were flanked by *loxP* sites to enable marker recycling by using Cre recombinase. For marker recycling, cells were transformed with 300 ng of circular pKTAC_Cre_hph encoding the Cre recombinase (45). Antibiotic selection markers needed for selection of diploid cells during mating experiments were introduced by integration of empty pPUZZLE expression vectors (46) into the *GAP* promoter or the *AOX1* terminator locus. Transformation of K. phaffii was performed by electroporation with either 500 ng of each fragment of a knockout cassette or 1 μg of linearized expression vector (40).

### Cultivation conditions.

Yeast cells were grown in standard YP medium (10 g/liter yeast extract, 20 g/liter soy peptone) containing 2% glucose as a carbon source. All liquid cultures were grown at 25°C. For selection of positive transformants, yeast extract-peptone-dextrose (YPD) agar supplemented with the appropriate antibiotic (50 μg/ml zeocin, 500 μg/ml Geneticin, 100 μg/ml nourseothricin, or 200 μg/ml hygromycin B) was used. Increased antibiotic concentrations of up to 100 μg/ml zeocin, 300 μg/ml hygromycin B, and 1 mg/ml Geneticin were used for the cultivation of diploid cells.

### Genomic DNA extraction and PCR.

Genomic DNA was extracted from overnight cultures using the Wizard genomic DNA purification kit (Promega) according to the manufacturer's protocol. All PCRs were performed with the Q5 High-Fidelity DNA polymerase (New England BioLabs). To determine the mating type of the cells, the genes in *MAT* locus 1 were determined by PCR using a touchdown PCR protocol. The annealing temperature was decreased from 70 to 60°C in 1°C steps for the first 10 cycles, followed by 25 cycles at 60°C. The elongation time was 150 s. For this analysis, the primers MAT_locus1_preDIC1_fwd, MAT_locus1_MATa_rev, and MAT_locus1_MATα_rev were mixed in the same reaction mixture.

### Mating protocols.

Qualitative mating experiments were performed similarly to a previously published procedure (19). Parallel streaks on YPD agar were made directly from glycerol stocks of the strains to be crossed. After approximately 24 h of incubation at 30°C, the cells were replicated twice at a 90° angle onto a mating agar plate (0.5% sodium acetate, 1% potassium chloride, 1% glucose, 2% agar) and incubated at 25°C for 3 days. For the selection of diploid cells, the cultures were replicated onto YPD agar plates containing the appropriate antibiotics. Diploid colonies at crossing points were picked and restreaked after 3 days at 30°C. Sporulation was induced by replicating the diploid cells onto mating agar and incubation at 25°C for 3 days. Diethyl ether extraction of the spores was performed according to a random spore analysis protocol described previously (19).

For the semiquantitative mating assay, nonselective YPD medium was inoculated with fresh colonies grown on YPD agar. After 18 to 20 h, approximately 6.5 × 10^7^ cells of each strain were mixed with their mating partner carrying a different antibiotic marker gene, plated on mating agar plates, and incubated at 25°C for 3 days. Cell numbers were estimated by measuring the optical density at 600 nm (OD_600_), with an OD_600_ of 1 corresponding to 1 × 10^7^ cells. The mated cells were washed from the plates with phosphate-buffered saline (PBS) (0.24 g/liter KH_2_PO_4_, 1.8 g/liter Na_2_HPO_4_ · 2 H_2_O, 0.2 g/liter KCl, 8 g/liter NaCl), and appropriate dilutions were plated on YPD agar containing either one or both antibiotics used for the selection of diploid cells. Colonies were counted after 3 days at 30°C. The mating efficiency was calculated as the percentage of cells growing on the plates containing both antibiotics in relation to the number of cells growing in the presence of one of the antibiotics (using the cell count on the antibiotic plates with the lower number of cells).

### Microscopy.

For the analysis of sporulation, cells were mated and sporulated as described above. Sporulating cells were taken from agar plates and suspended in a drop of water on a Polysine microscopy slide (Thermo Scientific). The cells were then visualized using a Zeiss Axio Observer.Z1/7 microscope with a LCI Plan-Neofluar 63× water immersion objective (numerical aperture, 1.3) in bright-field mode. Images were processed with Zen 2.3 lite (blue edition) software (Carl Zeiss Microscopy GmbH).

### RNA extraction and quantitative PCR.

Three colonies of each strain were cultivated in YPD medium in shake flasks until OD_600_ values of 7 to 11 were reached. These cultures were used to inoculate mating medium (0.5% sodium acetate, 1% potassium chloride, 1% glucose) at a starting OD_600_ of 2. Samples for RNA extraction were taken at the end of the YPD culture and after 24 h in mating medium. Aliquots were harvested by centrifugation at full speed at 4°C. The cell pellets were immediately resuspended in 1 ml TRI reagent solution (Invitrogen) and stored at −70°C until further use. After mechanical cell disruption using glass beads, RNA was isolated according to the TRI reagent protocol. RNA concentrations and integrity were analyzed with a Nanodrop spectrophotometer and a Bioanalyzer (Agilent) using the RNA 6000 Nano kit. After DNase treatment with the Ambion DNA-free kit (Invitrogen), cDNA was synthesized using oligo(dT)_23_ primers (NEB) and the DyNAmo cDNA synthesis kit (Thermo Fisher Scientific). Quantitative PCR was performed using the Sensi Mix SYBR Hi-Rox kit (Bioline) on a Rotor-Gene Q instrument (Qiagen). Purified PCR products of the analyzed genes were used to generate standard curves for quantification. Changes in transcript levels in *dic1-2*Δ *mat*Δ strains were calculated relative to the corresponding *dic1-2*Δ control using the threshold cycle (ΔΔ*CT*) method. Transcript levels were normalized to *ACT1* (PP7435_Chr3-0993) expression.

### DNA content analysis.

For DNA staining, cells were fixed with 70% ethanol. Before staining, the cells were washed with PBST (PBS with Tween 20, 1:1,000) and incubated with RNase A (1 mg/ml) for 1 h. After another washing step with PBST, the cells were resuspended in PBS and sonicated to avoid clumping of the cells. Propidium iodide at a final concentration of 100 μM was added to the treated cells immediately before analysis on a Gallios flow cytometer (Beckman Coulter). Data were analyzed with Kaluza analysis software (Beckman Coulter).

## Supplementary Material

Supplemental material
