# Supplementary material for: Creation of Stable Heterothallic Strains of Komagataella phaffii Enables Dissection of Mating Gene Regulation
Source: Mol Cell Biol. 2017 Dec 29;38(2):e00398-17. doi: 10.1128/MCB.00398-17 (PMC5748462; doi:10.1128/MCB.00398-17)
Supplement: Supplemental material [file supp_38_2_e00398-17__index.html]

Supplemental material 

# Creation of Stable Heterothallic Strains of Komagataella phaffii Enables Dissection of Mating Gene Regulation

## Supplemental material

- Supplemental file 1 -

  Fig. S1 (Mating plates) and S2 (Generation of split marker cassettes for gene deletions) and Tables S1 (*P* values for comparison of expression levels in mating medium), S2 (*P* values for comparison of expression levels in YPS versus mating medium), S3 (*MAT* and *STE2*/*STE3* transcript levels in *dic1-2*Δ *mat*Δ strains), and S4 (Primers)

  PDF, 2.2M
