# Supplementary material for: Creation of Stable Heterothallic Strains of Komagataella phaffii Enables Dissection of Mating Gene Regulation
Source: Mol Cell Biol. 2017 Dec 29;38(2):e00398-17. doi: 10.1128/MCB.00398-17 (PMC5748462; doi:10.1128/MCB.00398-17)
Supplement: Supplemental material [file MCB.00398-17_zmb002181679s1.pdf]

Creation of stable heterothallic strains of *Komagataella phaffii* enables dissection of mating gene regulation

Lina Heistinger, Brigitte Gasser, Diethard Mattanovich

Supplemental material

FIG S1 Mating plates.

A

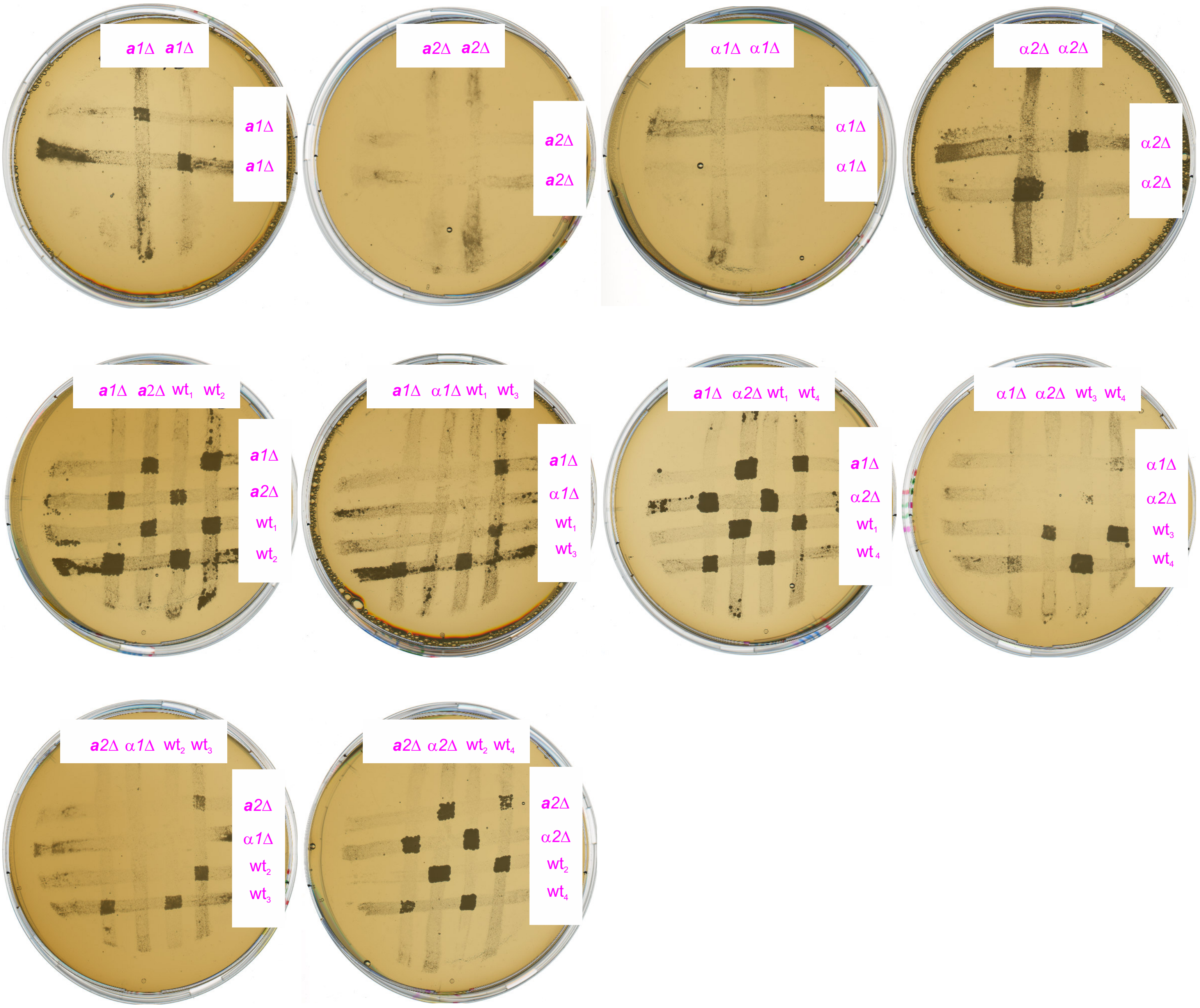

B

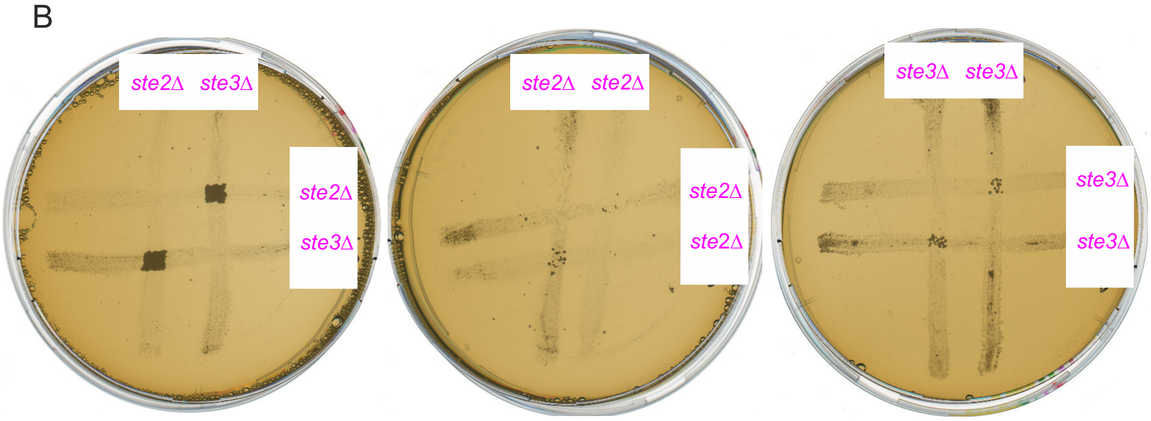

C

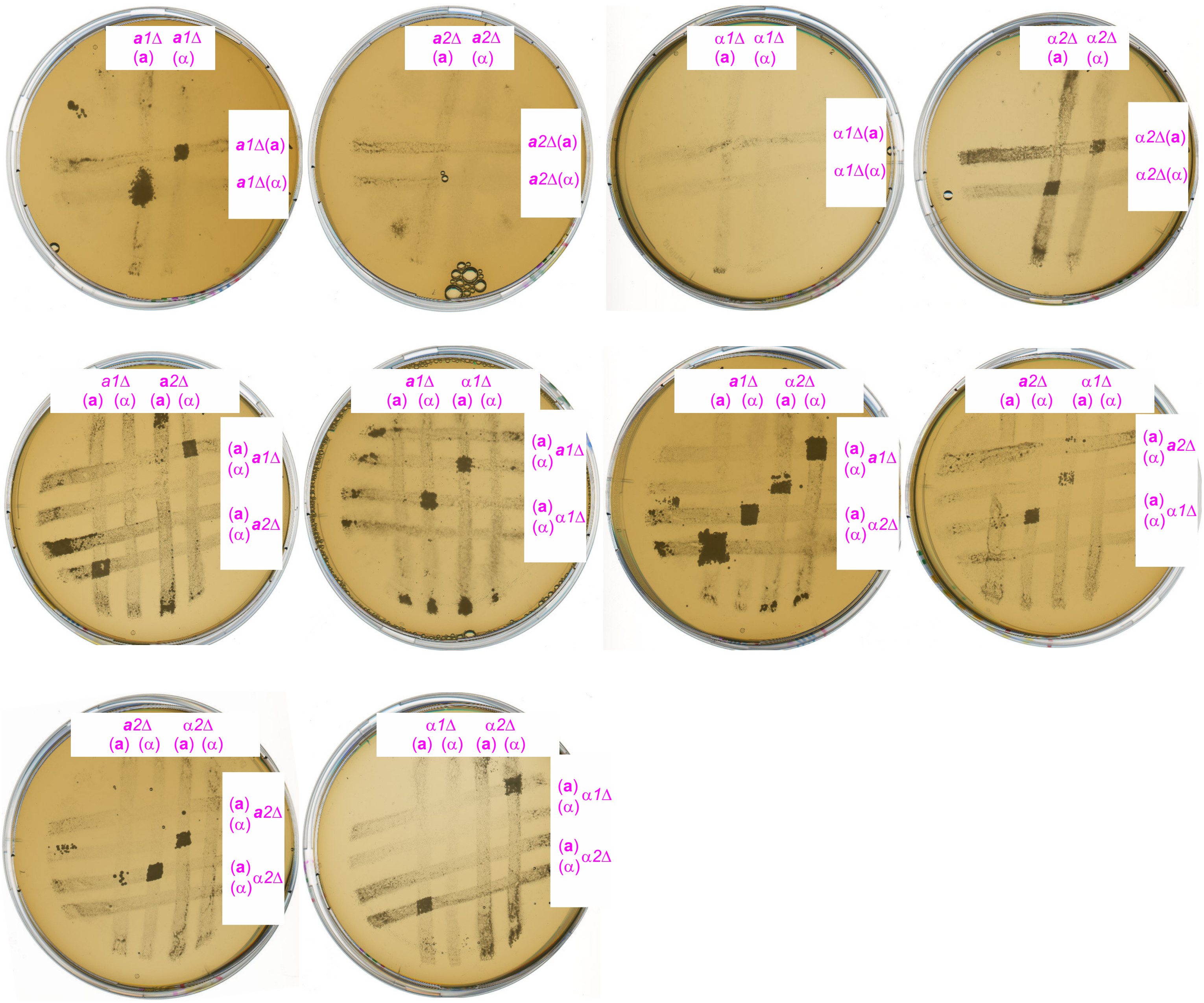
a1Δ a1Δ
(a) (α)
a1Δ(a)
a1Δ(α)
a2Δ a2Δ
(a) (α)
a2Δ(a)
a2Δ(α)
α1Δ α1Δ
(a) (α)
α1Δ(a)
α1Δ(α)
α2Δ α2Δ
(a) (α)
α2Δ(a)
α2Δ(α)
a1Δ a2Δ
(a) (α) (a) (α)
(a) (α) a1Δ
(a) (α) a2Δ
a1Δ α1Δ
(a) (α) (a) (α)
(a) (α) a1Δ
(a) (α) α1Δ
a1Δ α2Δ
(a) (α) (a) (α)
(a) (α) a1Δ
(a) (α) α2Δ
a2Δ α1Δ
(a) (α) (a) (α)
(a) (α) a2Δ
(a) (α) α1Δ
a2Δ α2Δ
(a) (α) (a) (α)
(a) (α) a2Δ
(a) (α) α2Δ
α1Δ α2Δ
(a) (α) (a) (α)
(a) (α) α1Δ
(a) (α) α2Δ

D

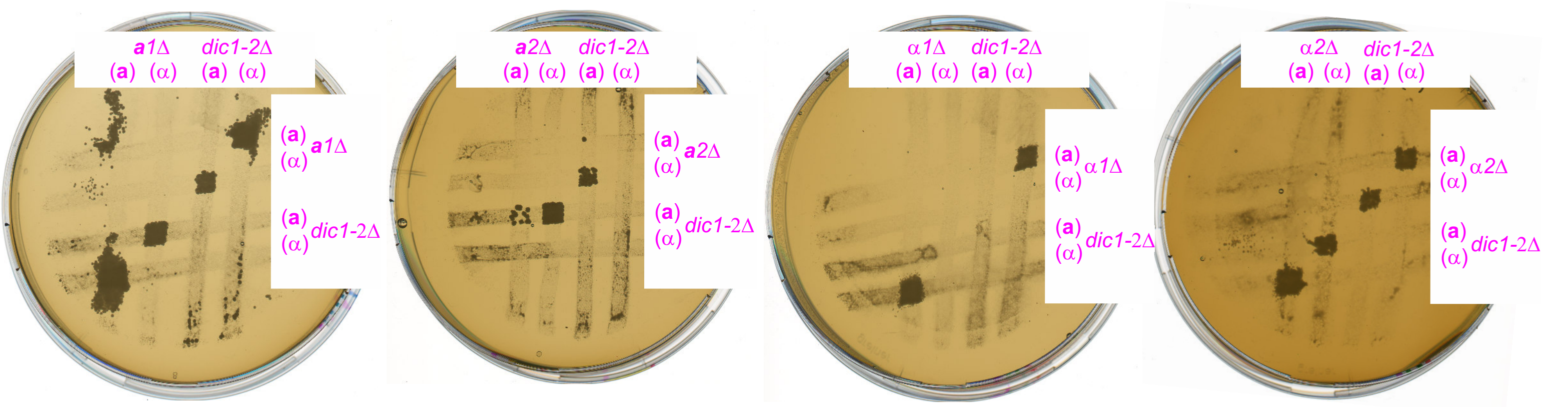
a1Δ dic1-2Δ
(a) (α) (a) (α)
(a) (α) a1Δ
(a) (α) dic1-2Δ
a2Δ dic1-2Δ
(a) (α) (a) (α)
(a) (α) a2Δ
(a) (α) dic1-2Δ
α1Δ dic1-2Δ
(a) (α) (a) (α)
(a) (α) α1Δ
(a) (α) dic1-2Δ
α2Δ dic1-2Δ
(a) (α) (a) (α)
(a) (α) α2Δ
(a) (α) dic1-2Δ

FIG S1 Mating plates. To analyse the effect of different mutations, strains were crossed on mating agar and replicated onto selective agar plates to evaluate diploid cell formation. Representative images of all tested combinations are shown. To cross two strains with the same mutation, additional marker genes were integrated for selection. (A) Mating of homothallic *mat*Δ strains with each other and the wildtype. Please note that the two wildtype strains on each plate carry different antibiotic markers for the selection of diploid clones with both mutant strains present on the same plate (1-ZeoR, 2-hphMX, 3-kanMX, 4-NatMX). (B) Mating of *ste2*Δ and *ste3*Δ strains. (C) Mating of heterothallic *dic1-2*Δ *mat*Δ strains. (D) Mating of heterothallic *dic1-2*Δ *mat*Δ strains with the unmodified *dic1-2*Δ strains.

Table S1 p-values for comparison of expression levels in mating medium

gene \ strains	*dic1-2*Δ(α)/*dic1-2*Δ(**a**)	*dic1-2*Δ(α)/wt	*dic1-2*Δ(**a**)/wt
*MAT***a***1*	0.0018	0.0235	0.1377
*MAT***a***2*	0.0061	0.0212	0.0056
*MAT*α*1*	0.0057	0.1714	0.0047
*MAT*α*2*	0.0001	0.0935	0.0055
STE2	0.0036	0.0117	0.0003
STE3	0.0037	0.0043	0.0019

All p-values were calculated from three biological replicates using a Student's t-test.

Table S2 p-values for comparison of expression levels in YPD vs. mating medium

gene \ strain	*dic1-2*Δ(α)	*dic1-2*Δ(**a**)	wildtype
*MAT***a***1*	0.0356	0.0127	0.0460
*MAT***a***2*	-	0.0064	0.0209
*MAT*α*1*	0.0058	-	0.0047
*MAT*α*2*	0.0001	-	0.0058
STE2	-	0.0036	0.0109
STE3	0.0033	0.0074	0.0018

All p-values were calculated from three biological replicates using a Student's t-test. Values are not shown for comparisons in which transcript levels were extremely low in both conditions.

Table S3 Relative *MAT* and *STE2* / *STE3* transcript levels in *dic1-2*Δ *mat*Δ strains.

	*MAT**a**1*			*MAT**a**2*			*MATα1*			*MATα2*			*STE2*			*STE3*		
strain	fold change	SD	p-value	fold change	SD	p-value	fold change	SD	p-value	fold change	SD	p-value	fold change	SD	p-value	fold change	SD	p-value
*dic1-2*Δ(**a**)	**1.0**	+/- 0.10	-	**1.0**	+/- 0.16	-	1.0	+/- 0.08	-	1.0	+/- 0.31	-	**1.0**	+/- 0.16	-	1.0	+/- 0.19	-
*mat**a**1*Δ(**a**)	**-**	-	-	**1.16**	+/- 0.15	0.1282	0.95	+/- 0.23	0.7183	0.66	+/- 0.17	0.0494	**1.14**	+/- 0.44	0.7589	1.20	+/- 0.26	0.2013
*mat**a**2*Δ(**a**)	**1.76**	+/- 0.05	0.0000	**-**	-	-	0.60	+/- 0.08	0.0001	0.67	+/- 0.41	0.1669	**0.01**	+/- 0.00	0.0000	0.19	+/- 0.03	0.0000
*matα1*Δ(**a**)	**1.05**	+/- 0.28	1.0000	**1.27**	+/- 0.33	0.2324	-	-	-	6.01	+/- 0.48	0.0000	**1.91**	+/- 0.50	0.0049	1.12	+/- 0.25	0.4547
*matα2*Δ(**a**)	**1.35**	+/- 0.18	0.0022	**1.10**	+/- 0.29	0.6250	1.08	+/- 0.04	0.0022	-	-	-	**1.40**	+/- 0.23	0.0056	2.02	+/- 0.20	0.0000
*dic1-2*Δ(α)	1.0	+/- 0.14	-	1.0	+/- 0.17	0.0596	**1.0**	+/- 0.21	-	**1.0**	+/- 0.20	-	1.0	+/- 0.13	-	**1.0**	+/- 0.22	-
*mat**a**1*Δ(α)	-	-	-	0.78	+/- 0.20	-	**1.10**	+/- 0.06	0.3423	**1.25**	+/- 0.15	0.0486	1.12	+/- 0.16	0.2168	**1.11**	+/- 0.07	0.3015
*mat**a**2*Δ(α)	1.37	+/- 0.07	0.0045	-	-	-	**1.04**	+/- 0.08	0.7193	**1.12**	+/- 0.07	0.2217	0.89	+/- 0.14	0.2354	**0.98**	+/- 0.10	0.8228
*matα1*Δ(α)	0.75	+/- 0.27	0.0724	0.44	+/- 0.20	0.0045	**-**	-	-	**1.44**	+/- 0.37	0.0339	1.03	+/- 0.21	0.9094	**0.01**	+/- 0.00	0.0000
*matα2*Δ(α)	0.86	+/- 0.16	0.1329	0.94	+/- 0.26	0.5183	**0.46**	+/- 0.08	0.0000	**-**	-	-	1.10	+/- 0.06	0.1536	**1.00**	+/- 0.06	0.9808

Changes in *MAT* and *STE2 / STE3* transcript levels in the different *dic1-2*Δ *mat*Δ strains under mating conditions were analyzed by quantitative PCR. Gene expression was normalized to *ACT1* and is given as fold change to the expression in the respective unmodified *dic1-2*Δ strain. Data was analyzed using the $\Delta\Delta C_T$ method and fold changes were calculated as $2^{-\Delta\Delta CT}$. Fold change values of highly expressed genes transcribed from the active *MAT* locus are indicated in bold. P-values were calculated using a Student's t-test.

FIG S2 Generation of split marker cassettes for gene deletions

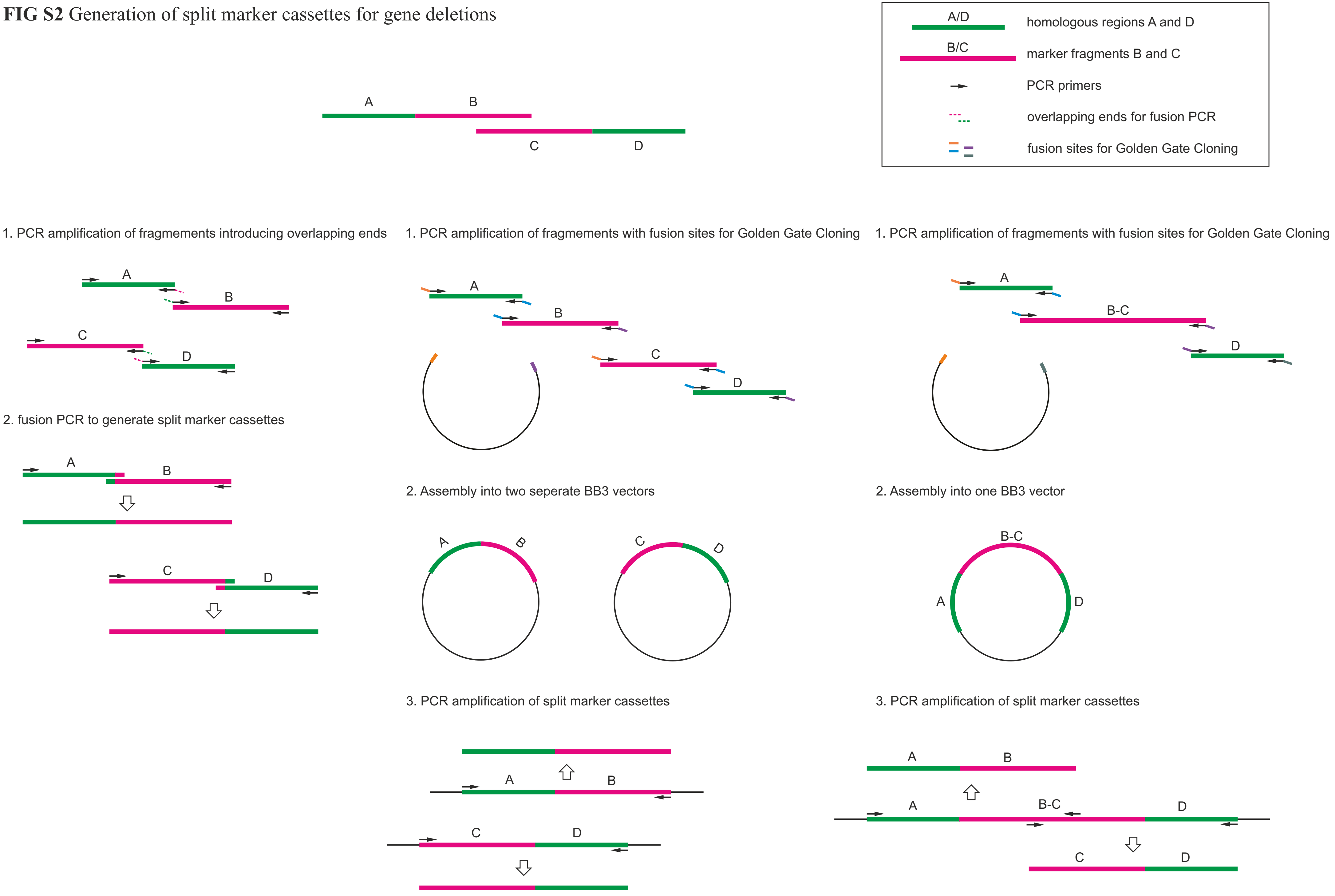

FIG S2 Schematic of different strategies for the generation of the split-marker knockout cassettes. Generally, the knockout cassettes consisted of the homologous region for genomic integration (fragment A + D shown in green) and two halves of an antibiotic resistance marker with overlapping ends of around 440 bp (B + C shown in magenta). In the first approach, the separate fragments were amplified by PCR introducing small overlapping sequences and the final cassettes were generated by a second fusion PCR step. For the two remaining strategies, the fragments were introduced into plasmid vectors by Golden Gate Cloning. PCR amplified fragments carrying the required fusion sites were either introduced into separate vectors for the two halves of the split marker cassette or cloned as the full knockout cassette containing the whole marker sequence. In both cases the final split-marker fragments were amplified by PCR using the purified vectors as templates.

Table S4 List of primers used in this study.

**knockout cassettes assembled by fusion PCR of A-B and C-D**		
MATa1	homologous region A	AACTTGCTCTCCTCGATTTG
		GTTGTCGACCTGCAGCGTACCTTGGGTCAGATGTGCGT
	marker fragment B	ACGCACATCTGACCCAAGGTACGCTGCAGGTCGACAAC
		CTGCTCGCCGATCTCGGTC
	marker fragment C	TGACCAGTGCCGTTCCGG
		TTACGTTATGGATGGGACTAGTGGATCTGATATCACCTA
	homologous region D	TAGGTGATATCAGATCCACTAGTCCCATCCATAACGTAAC
		CCGTGATGTAGCCGAAGAG
MATα1+2	homologous region A	GTGATGTAGCCGAAGAGT
		GTTGTCGACCTGCAGCGTACATCCTGGAATGAGCTACT
	marker fragment B	AGTAGCTCATTCCAGGATGTACGCTGCAGGTCGACAAC
		CGGTGAGAATGGCAAAAGCTTATG
	marker fragment C	AAGCCCGATGCGCCAGAGTTG
		CAGTTTTCATGTCTCAAGCGAGTGGATCTGATATCACCTA
	homologous region D	TAGGTGATATCAGATCCACTCGCTTGAGACATGAAAACTG
		CCAGCCAAACAAACACAATC
**knockout cassettes assembled into 2 different vectors by Golden Gate Cloning (A-B and C-D)**		
MATa2	homologous region A (fusion sites A-B)	GGTCTCC**GATC**TTTTGGTATGGAGGTGCCG
		GGTCTCC**CCGG**ACAACGTTTAGCTGCCTTC
	marker fragment B (fusion sites B-C)	GGTCTCC**CCGG**AGTGGATCTGATATCACCTA
		GGTCTCC**AATT**AGACCTGCCTGAAACCGAACTGC
	marker fragment C (fusion sites A-B)	GGTCTCC**GATC**AGATGTTGGCGACCTCGTATTG
		GGTCTCC**CCGG**GTACGCTGCAGGTCGACAAC
	homologous region D (fusion sites B-C)	GGTCTCC**CCGG**AATTCTATCCATCAGACCTG
		GGTCTCC**AATT**TGCCTGTTGCTTCTATACT
MATα1	homologous region A (fusion sites A-B)	GGTCTCC**GATC**GCTCAGCGACCAAGAAAA
		GGTCTCC**CCGG**AGCTACTCAGCACTAGAAA
	marker fragment B (fusion sites B-C)	GGTCTCC**CCGG**GTACGCTGCAGGTCGACAAC
		GGTCTCC**AATT**CGGTGAGAATGGCAAAAGCTTATG
	marker fragment C (fusion sites A-B)	GATAGGTCTCC**GATC**AAGCCCGATGCGCCAGAGTTG
		GATAGGTCTCC**CCGG**AGTGGATCTGATATCACCTA
	homologous region D (fusion sites B-C)	GATAGGTCTCC**CCGG**TATCTATCACATGCACGCAC
		GATAGGTCTCC**AATT**TTCCATCCTTTATTCGCCT
MATα2	homologous region A (fusion sites A-B)	GGTCTCC**GATC**CTCTACAGAATGCGCGAA
		GGTCTCC**CCGG**ACAGAGCTACTTCAAGCA
	marker fragment B (fusion sites B-C)	GGTCTCC**CCGG**GTACGCTGCAGGTCGACAAC
		GGTCTCC**AATT**TGCGTTGACGTTGGTGAC
	marker fragment C (fusion sites A-B)	GGTCTCC**GATC**GTCCTTCACCACCGACACC
		GGTCTCC**CCGG**AGTGGATCTGATATCACCTA
	homologous region D (fusion sites B-C)	GGTCTCC**CCGG**AACTGATAAGAATCGCGG
		GGTCTCC**AATT**CCAGCCAAACAAACACAA
**knockout cassettes assembled into 1 vector by Golden Gate Cloning (A-D)**		
MATa1+2	homologous region A (fusion sites A-B)	GATAGGTCTCC**GATC**TTTTGGTATGGAGGTGCCG
		GATAGGTCTCC**CCGG**ACAACGTTTAGCTGCCTTC

	marker fragment B-C (fusion sites B-C)	GATAGGTCTCC**CCGG**GTACGCTGCAGGTCGACAAC
		GATAGGTCTCC**AATT**AGTGGATCTGATATCACCTA
	homologous region D (fusion sites C-D)	GATAGGTCTCC**AATT**AGTCCCATCCATAACGTAAG
		GATAGGTCTCC**AGCT**CCGTGATGTAGCCGAAGAG
STE2	homologous region A (fusion sites A-B)	GATAGGTCTCC**GATC**AAATCTTCAGCAAGAACGG
		GATAGGTCTCC**CCGG**GCAGGCAATAATCAAACGG
	marker fragment B-C (fusion sites B-C)	GATAGGTCTCC**CCGG**GTACGCTGCAGGTCGACAAC
		GATAGGTCTCC**AATT**AGTGGATCTGATATCACCTA
	homologous region D (fusion sites C-D)	GATAGGTCTCC**AATT**GCACGTTCACTTCTCTTTTACT
		GATAGGTCTCC**AGCT**ACTGAAACACCAAAAGATCCC
STE3	homologous region A (fusion sites A-B)	GATAGGTCTCC**GATC**GGAAGCCAAGGGGAATGA
		GATAGGTCTCC**CCGG**GATAAAGTGTGACGCGGA
	marker fragment B-C (fusion sites B-C)	GATAGGTCTCC**CCGG**GTACGCTGCAGGTCGACAAC
		GATAGGTCTCC**AATT**AGTGGATCTGATATCACCTA
	homologous region D (fusion sites C-D)	GATAGGTCTCC**AATT**AACAGGCCAAACATGAAGA
		GATAGGTCTCC**AGCT**CTCGCATTAACCATTTTCTCC
DIC1-2	homologous region A (fusion sites A-B)	GATAGGTCTCC**GATC**TGAAAGATTTGGGTTGCG
		GATAGGTCTCC**CCGG**GATGATGTTCCTGGTTTACT
	marker fragment B-C (fusion sites B-C)	GATAGGTCTCC**CCGG**GTACGCTGCAGGTCGACAAC
		GATAGGTCTCC**AATT**AGTGGATCTGATATCACCTA
	homologous region D (fusion sites C-D)	GATAGGTCTCC**AATT**CATCATCGTTTTCCCACAAG
		GATAGGTCTCC**GAGA**CTCTGGAGGTATATG
		GATAGGTCTCC**TCTC**AAGGAGCAATCATACC
		GATAGGTCTCC**AGCT**TCCCAAATACGCACACAG
***MATa1+2* integration**	homologous region A (fusion sites A-B)	GATAGGTCTCC**GATC**TAACGGGGATCATGTATCGG
		GATAGGTCTCC**CCGG**TTATTCATCCGCATGGTATCCA
	marker fragment B-C (fusion sites B-F)	GATAGGTCTCC**CCGG**GTACGCTGCAGGTCGACAAC
		GATAGGTCTCC**AAGC**AGTGGATCTGATATCACCTA
	MATa1+2 (fusion sites F-C)	GATAGGTCTCC**GCTT**GTAACTGAGTTGAATAGCTTACG
		GATAGGTCTCC**AATT**CATTTAGTATTTGACGCTACAGG
	homologous region D (fusion sites C-D)	GATAGGTCTCC**AATT**CATCATCGTTTTCCCACAAG
		GATAGGTCTCC**GAGA**CTCTGGAGGTATATG
		GATAGGTCTCC**TCTC**AAGGAGCAATCATACC
		GATAGGTCTCC**AGCT**TCCCAAATACGCACACAG
***MATα1+2* integration**	homologous region A and *MATα1+2* (fusion sites A-B)	GATAGGTCTCC**GATC**TTTTGGTATGGAGGTGCCG
		GATAGGTCTCC**CCGG**GCAGATTATTAATGAAGTATAGAAACC
	marker fragment B-C (fusion sites B-C)	GATAGGTCTCC**CCGG**GTACGCTGCAGGTCGACAAC
		GATAGGTCTCC**AATT**AGTGGATCTGATATCACCTA
	homologous region D (fusion sites C-D)	GATAGGTCTCC**AATT**TTACTCTTCGTCCGCATGGT
		GATAGGTCTCC**AGCT**TAACGGGGATCATGTATCGG
**primers for cloning of homology templates for Cas9 mediated integration**		
MATa1	homologous region A (fusion sites A-B)	GATAGGTCTCC**GATC**AAGGCAGCTAAACGTTGT
		GATAGGTCTCC**CCGG**TGCCAAGACTTGTGTTTCT
	marker fragment B-C (fusion sites B-C)	GATAGGTCTCC**CCGG**GTACGCTGCAGGTCGACAAC
		GATAGGTCTCC**AATT**AGTGGATCTGATATCACCTA
	homologous region D (fusion sites C-D)	GATAGGTCTCC**AATT**TTACACAAAGCCAGCAAC

		GATAGGTCTCC**AGCT**ATGGGACTGAGCTTGAAG
MATa2	homologous region A (fusion sites A-B)	GATAGGTCTCC**GATC**AAGGCAGCTAAACGTTGT
		GATAGGTCTCC**CCGG**TGCCAAGACTTGTGTTTCT
	marker fragment B-C (fusion sites B-C)	GATAGGTCTCC**CCGG**GTACGCTGCAGGTCGACAAC
		GATAGGTCTCC**AATT**AGTGGATCTGATATCACCTA
	homologous region D (fusion sites C-D)	GATAGGTCTCC**AATT**CTAACTAGTTATCATACTTCTGTCCTTTCTACC
		GATAGGTCTCC**AGCT**TGTTTTCCCGAATAGGCT
MATα1	homologous region A (fusion sites A-B)	GATAGGTCTCC**GATC**ATTTCTAGTGCTGAGTAGC
		GATAGGTCTCC**CCGG**CCTTTCAAGTGTTCCCAA
	marker fragment B-C (fusion sites B-C)	GATAGGTCTCC**CCGG**GTACGCTGCAGGTCGACAAC
		GATAGGTCTCC**AATT**AGTGGATCTGATATCACCTA
	homologous region D (fusion sites C-D)	GATAGGTCTCC**AATT**CTAACTAGTTAACTCCGATTGCCTTTTGT
		GATAGGTCTCC**AGCT**GAATGCTCAAGAACGACG
MATα2	homologous region A (fusion sites A-B)	GATAGGTCTCC**GATC**ACTCCGATTGCCTTTTG
		GATAGGTCTCC**CCGG**GAGAAAAAGAAGGTTGCC
	marker fragment B-C (fusion sites B-C)	GATAGGTCTCC**CCGG**GTACGCTGCAGGTCGACAAC
		GATAGGTCTCC**AATT**AGTGGATCTGATATCACCTA
	homologous region D (fusion sites C-D)	GATAGGTCTCC**AATT**TTCCAACATTAAAGCGATCG
		GATAGGTCTCC**TAAC**TAGTTAGAGAATCTTGCAAGGGATATC
		GATAGGTCTCC**GTTA**CATCAGAGTTTTGTGCACA
		GATAGGTCTCC**AGCT**GCTACAGAAAACCGCGA
**primers for assembly of gRNAs by fusion PCR**		
for all gRNAs	CR_rib_1_gRNAall_rev	CGCCATGCCGAAGCATGTTGCCCAGCCGGCGCCAGCGAGGAGGCTGGGACCATGCCGGCC
	CR_rib_2_gRNAall.rev	GATAGGTCTCC**AAGC**GTCCAAAGCTGTCCCATTCGCCATGCCGAAGCATGTTGCCCAGCC
	CR_rib_3_gRNAall_rev	AGGCTGGGACCATGCCGGCCAAAAGCACCGACTCGGTGCCACTTTTTCAAGTTGATAACG
	CR_rib_4_gRNAall_fwd	ACTTTTTCAAGTTGATAACGGACTAGCCTTATTTTAACTTGCTATTTCTAGCTCTAAAAC
MATa1	MATa1_gRNA_1_fwd	GATAGGTCTCC**CATG**ATGATACTGATGAGTCCGTGAGGACGAAACGAGTAAGCTCGTCTA
	MATa1_gRNA_2_fwd	AAACGAGTAAGCTCGTCTATCATGGACAATAGGCATGGTTTTAGAGCTAGAAATAGCAAG
MATa2	MATa2_gRNA_1_fwd	GATAGGTCTCC**CATG**ATTCTGCTGATGAGTCCGTGAGGACGAAACGAGTAAGCTCGTCCA
	MATa2_gRNA_2_fwd	AAACGAGTAAGCTCGTCCAGAATCAAAGGATGACGATGTTTTAGAGCTAGAAATAGCAAG
MATα1	MATalpha1_gRNA1_fwd	GATAGGTCTCC**CATG**TTGCGGCTGATGAGTCCGTGAGGACGAAACGAGTAAGCTCGTCCC
	MATalpha1_gRNA2_fwd	AAACGAGTAAGCTCGTCCCGCAACCCAAATCTTTCAAGTTTTAGAGCTAGAAATAGCAAG
MATα2	MATalpha2_gRNA_1_fwd	GATAGGTCTCC**CATG**TTTCTCCTGATGAGTCCGTGAGGACGAAACGAGTAAGCTCGTCGA
	MATalpha2_gRNA_2_fwd	AAACGAGTAAGCTCGTCGAGAAAAAGAAGGTTGCCAAGTTTTAGAGCTAGAAATAGCAAG
**primers for cassette amplification and integration control**		
MATa1	MATa1_control_fwd	TGTAAGAGGAGAGTGGAATTG
	MATa1_control_rev	AGGGAGTCGTAAGATTGG

MATa2	MATa2_ampl_fwd	TTTTGGTATGGAGGTGCCG
	HphMX_rev	AGATGTTGGCGACCTCGTATTG
	HphMX_fwd	AGACCTGCCTGAAACCGAACTGC
	MATa2_ampl_rev	TGCCTGTTGCTTCTATACT
	MATa2_control_fwd	CAATCCTCCATGACTCAGA
	MATa2_control_rev	ATAACGAGATGACTGACGG
MATa1+2	MATa12_ampl_fwd	TTTTGGTATGGAGGTGCCG
	Zeo_rev	CTGCTCGCCGATCTCGGTC
	Zeo_fwd	TGACCAGTGCCGTTCCGG
	MATa12_ampl_rev	CCGTGATGTAGCCGAAGAG
	MATa12_control_fwd = Zeo_fwd	TGACCAGTGCCGTTCCGG
	MATa12_control_rev = MATa1_control_rev	AGGGAGTCGTAAGATTGG
MATα1	MATα1_ampl_fwd	GCTCAGCGACCAAGAAAA
	KanMX_rev	CGGTGAGAATGGCAAAAGCTTATG
	KanMX_fwd	AAGCCCGATGCGCCAGAGTTG
	MATα1_ampl_rev	TTCCATCCTTTATTCGCCT
	MATα1_control_fwd	GTGATGTAGCCGAAGAGT
	MATα1_control_rev	AGAATGCTCAAGAACGAC
MATα2	MATα2_ampl_fwd	CTCTACAGAATGCGCGAA
	NatMX_rev	TGCGTTGACGTTGGTGAC
	NatMX_fwd	GTCCTTCACCACCGACACC
	MATα2_ampl_rev	CCAGCCAAACAAACACAA
	MATα2_control_fwd	CTAGTGCGAGGAGTTTCT
	MATα2_control_rev	CTTCTTCTCGTTTCCCACC
MATα1+2	MATα12_control_fwd	GTAAGGGAGTCGTAAGATTGG
	MATα12_control_rev	AGTGCAGGAGGAAACAAG
STE2	STE2_ampl_fwd	AAATCTTCAGCAAGAACGG
	KanMX_rev	CGGTGAGAATGGCAAAAGCTTATG
	KanMX_fwd	AAGCCCGATGCGCCAGAGTTG
	STE2_ampl_rev	ACTGAAACACCAAAAGATCCC
	STE2_control_fwd	CGACAGAAGGGAAGCAAG
	STE2_control_rev	AAAAGAGATAACGTCCAGCG
STE3	STE3_ampl_fwd	GGAAGCCAAGGGGAATGA
	NatMX_rev	TGCGTTGACGTTGGTGAC
	NatMX_fwd	GTCCTTCACCACCGACACC
	STE3_ampl_rev	CTCGCATTAACCATTTTCTCC
	STE3_control_fwd	CCAAAATCCACCTTGATCCC
	STE3_control_rev	TACAAGACTCACCTTCTGACC
DIC1-2	DIC1_ampl_fwd	TGAAAGATTTGGGTTGCG
	KanMX_rev	CGGTGAGAATGGCAAAAGCTTATG
	KanMX_fwd	AAGCCCGATGCGCCAGAGTTG
	DIC1_ampl_rev	TCCCAAATACGCACACAG
	DIC1_control_fwd	CTGTGGGCGTATTGGGAA
	DIC1_control_rev	GTGTCTCGAGTTTTGTGTAGT
***MATa1+2* integration**	MATa12_integ_ampl_fwd	TAACGGGGATCATGTATCGG
	NatMX_rev	TGCGTTGACGTTGGTGAC
	NatMX_fwd	GTCCTTCACCACCGACACC

	MATa12_integ_ampl_fwd = DIC1_ampl_rev	TCCCAAATACGCACACAG
	MATa12_integ_control_fwd	AACGCAGGAGAACCGTAAAC
	MATa12_integ_control_rev = DIC1_control_rev	GTGTCTCGAGTTTTGTGTAGT
***MATα1+2* integration**	MATα12_integ_ampl_fwd	TTTTGGTATGGAGGTGCCG
	NatMX_rev	TGCGTTGACGTTGGTGAC
	NatMX_fwd	GTCCTTCACCACCGACACC
	MATα12_integ_ampl_rev	TAACGGGGATCATGTATCGG
	MATα12_integ_control_fwd	GGAATAAAGCGTACTGGTG
	MATα12_integ_control_rev	GCTCTACAGAATGCGCGAA
**primers for mating-type determination**		
***MAT* locus1**	MAT_locus1_preDIC1_fwd	GCATCCAGCATAATTTAGATT
	MAT_locus1_MATa_rev	AACTCTGGGATCTTTGGA
	MAT_locus1_MATα_rev	CGCTTGAGACATGAAAACTG

Fusion sites for Golden Gate Cloning are indicated in bold. Underlined characters indicate guide RNA sequences for CRISPR/Cas9 mediated homology-directed repair.
